# A feasibility study using motivational interviewing and a smartphone application to promote physical activity (+Stay-Active) for women with gestational diabetes

**DOI:** 10.1186/s12884-024-06508-w

**Published:** 2024-05-14

**Authors:** Ralph Smith, Rebecca Gould, Yvonne Kenworthy, Nerys Astbury, Iwan Smith, Jacqueline Birks, Paul Bateman, Jane E. Hirst, Susan Jebb, Moscho Michalopoulou, Richard Pulsford, Cristian Roman, Mauro Santos, Nicola Wango, Amy Wire, Lucy Mackillop

**Affiliations:** 1 Oxford University Hospitals NHS Foundation Trust, Oxford, UK; 2 Nuffield Department of Women’s & Reproductive Health, University of Oxford, Oxford, UK; 3 Nuffield Department of Primary Care Health Sciences, University of Oxford, Oxford, UK; 4 Centre for Statistics in Medicine, University of Oxford, Oxford, UK; 5 Cardiovascular Clinical Research Facility, University of Oxford, Oxford, UK; 6 Faculty of Health and Life Sciences, University of Exeter, Exeter, UK; 7 Department of Engineering Science, Institute of Biomedical Engineering, University of Oxford, Oxford, UK; 8 Independent Researcher, Patient Representative, Oxford, UK; 9 The George Institute for Global Health, Imperial College London UK, London, UK; 10 Independent Researcher, Oxford, UK

**Keywords:** Gestational diabetes mellitus, Physical activity, Smartphone applications

## Abstract

**Background:**

Physical activity (PA) interventions have an encouraging role in gestational diabetes mellitus (GDM) management. Digital technologies can potentially be used at scale to support PA. The aim of this study was to assess the feasibility and acceptability of + Stay-Active: a complex intervention which combines motivational interviewing with a smartphone application to promote PA levels in women with GDM.

**Methods:**

This non-randomised feasibility study used a mixed methods approach. Participants were recruited from the GDM antenatal clinic at Oxford University Hospitals. Following baseline assessments (visit 1) including self-reported and device determined PA measurements (wrist worn accelerometer), women participated in an online motivational interview, and then downloaded (visit 2) and used the Stay-Active app (Android or iOS). Women had access to Stay-Active until 36 weeks’ gestation, when acceptability and PA levels were reassessed (visit 3). The primary outcome measures were recruitment and retention rates, participant engagement, and acceptability and fidelity of the intervention. Secondary outcome measures included PA levels, app usage, blood glucose and perinatal outcomes. Descriptive statistics were performed for assessments at study visits. Statistics software package Stata 14 and R were used.

**Results:**

Over the recruitment period (46 weeks), 114 of 285 women met inclusion criteria and 67 (58%) enrolled in the study. Mean recruitment rate of 1.5 participants/clinic with 2.5 women/clinic meeting inclusion criteria. Fifty-six (83%) received the intervention at visit 2 and 53 (79%) completed the study. Compliance to accelerometer measurement protocols were sufficient in 78% of participants (52/67); wearing the device for more than 10 h on 5 or more days at baseline and 61% (41/67) at 36 weeks. There was high engagement with Stay-Active; 82% (55/67) of participants set goals on Stay-Active. Sustained engagement was evident, participants regularly accessed and logged multiples activities on Stay-Active. The intervention was deemed acceptable; 85% of women rated their care was satisfactory or above, supported by written feedback.

**Conclusions:**

This combined intervention was feasible and accepted. Recruitment rates were lower than expected. However, retention rates remained satisfactory and participant compliance with PA measurements and engagement was a high. Future work will explore the intervention’s efficacy to increase PA and impact on clinical outcomes.

**Trial registration:**

The study has received a favourable opinion from South Central—Hampshire B Research Ethics Committee; REC reference: 20/SC/0342. ISRCTN11366562.

**Supplementary Information:**

The online version contains supplementary material available at 10.1186/s12884-024-06508-w.

## Introduction

Gestational diabetes mellitus (GDM) is defined as any degree of glucose intolerance first detected during pregnancy [1]. There are serious associated complications for both mother and baby [2–4]. Glycaemic control is fundamental to GDM management [5]. Increasing blood glucose concentrations have been suggested as one of the main mechanisms for the increased risk of adverse maternal and infant outcomes [6]. Management interventions include blood glucose monitoring, lifestyle intervention and pharmacological therapy. Of those lifestyle interventions, only dietary modifications and physical activity (PA) have demonstrated possible health benefits for maternal and fetal outcomes [7].

Evidence supporting the benefits of PA amongst women with GDM is growing. Improvements in glycaemic control and reduced insulin requirements has been shown in meta-analyses of PA interventions amongst women with GDM [8, 9]. The National Institute for Health and Care Excellence (NICE), recommends women with GDM to exercise regularly, for example, walking for 30 min after a meal [10]. Women have highlighted their request for clear, simple and specific PA messages with accommodating options [11].

Behaviour Change Techniques (BCTs) are felt to be fundamental to successful PA interventions. A BCT is defined as the smallest “active ingredient” of an intervention. There are 93 internationally agreed and validated BCTs [12]. Techniques such as goal setting and action planning, shaping knowledge and comparison of outcomes have been effective in attenuating the observed decline of PA during pregnancy [13].

Our previous work has shown promise that motivational interviewing (using several BCTs) can help to increase PA in women with GDM [14]. Motivational interviewing was embedded into the routine clinical care for 64 women with GDM. Women were invited to a 20-min individual motivational interview focusing on increasing or maintaining PA during their pregnancy. A specific motivational interviewing framework was used. This included essential micro-skills such as individual goal setting, activity planning and specific information about the benefits and types of recommended PA. A significant increase in self-reported PA levels after two weeks was found [14]. Whilst motivational interviewing provides an initial catalyst for behaviour change, supporting these lifestyle changes remains challenging.

In the UK, many hospital trusts are using digital technologies to support remote monitoring and glycaemic control management [15]. Remote digital devices provide an enlightening prospect to support PA remotely. A smartphone application ‘Stay-Active’, (referred to as the ‘app’) was designed to enhance and support women following the existing motivational interviewing intervention. A systematic approach using the Behaviour Change Wheel (BCW) [16] underpinned the design of this multi-component application. Current evidence, focus groups and input from key stakeholders all informed the development process [17]. Stay-Active delivers ten BCTs through a bespoke educational resource centre, using goal setting and action planning features and tailored performance feedback with individualised messages. A distinctive feature is the clinicians’ ability to interact with the user. Recorded PA can be reviewed by clinicians remotely and specific tailored messages can be sent to users to support their PA levels. This study aimed to determine the feasibility and acceptability of the complex intervention + Stay-Active in women with GDM. + Stay Active combines an initial motivational interview with the smartphone application ‘Stay-Active’ to empower and support women; utilising PA in the management of GDM. This information will determine if a randomised control trial (RCT) to evaluate this intervention is feasible.

## Methods

The purpose of the study is to evaluate how women with GDM interact, engage with, and respond to a complex intervention, known as + Stay-Active. The study protocol has been previously published and contains a detailed description of the methods used, study outcomes and progression criteria [18].

### Study design

**Fig. 1:**
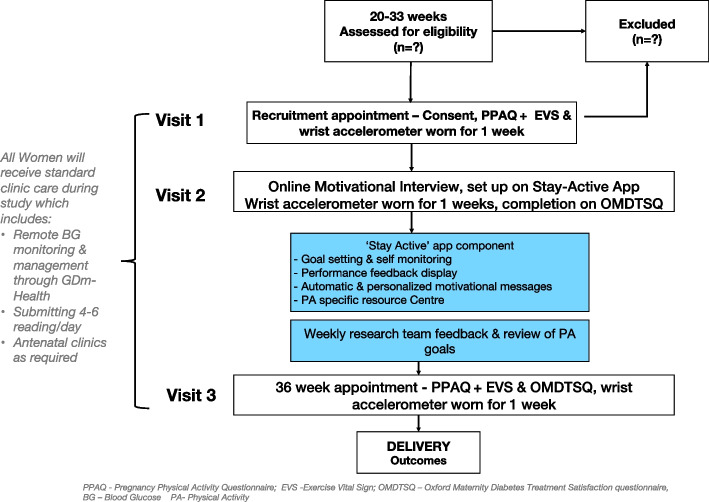
A flow chart of the study design

This feasibility study was a non-randomised single arm trial, with all participants receiving the + Stay-Active intervention. A mixed methods approach was used. Figure 1 illustrates a flow chart of the study design, visits, and assessments.

### Setting & study participants

All participants were recruited from National Health Service (NHS) maternity clinics at the Women’s Centre, Oxford University Hospitals NHS Foundation Trust. Pregnant women at least 20 weeks gestation with a confirmed diagnosed of GDM (defined by the testing method used in this NHS hospital at the time of recruitment) were eligible to take part. During recruitment, the diagnosis of GDM was as per the NICE Diabetes in Pregnancy 2015 guideline [19]. From April 2020, the unit adopted the Royal College of Obstetricians & Gynaecologists (UK) guidance [20] during the COVID-19 pandemic. From January 2022 the unit changed to use the NICE thresholds for the 75 g OGTT diagnosis [21]. Recruitment ran from April 2021 to April 2022.

#### Visit 1: Recruitment and baseline assessments

Women attending the GDM clinic who met the inclusion criteria (see Table 1) were identified by the clinical team at their appointment. Following their clinic appointment, women were invited to enrol on the study. Participants were then asked to complete a baseline assessment of PA using an online version of Pregnancy Physical Activity Questionnaire (PPAQ) [22] and the exercise vital sign assessment (EVS) [23]. Participants wore a tri-axial accelerometer (GENEActiv, Active Insights Ltd, Kimbolton, UK) on their non-dominant wrist for at least seven consecutive days (day and night). This duration of measurement has been shown to provide robust and reliable estimates of moderate to vigorous physical activity (MVPA) during pregnancy [24]. The GENEActiv accelerometer objectively measures and stores movement acceleration in g (the standard SI unit of acceleration) at a high frequency (100 Hz or 100 times per second) for offline analysis, thereby allowing a range of data processing techniques to be applied post data-collection to derive estimates of PA. Participants were provided with an instruction sheet which includes general care instructions.

**Table 1:** Study inclusion and exclusion criteria

**Inclusion criteria**	**Exclusion criteria**
Women who are more than 20 completed weeks pregnant and less than 33 completed weeks pregnant with a singleton pregnancy • Abnormal OGTT as defined by IADPSG, HbA1C, fasting plasma glucose or random blood glucose as defined by RCOG Guidance for maternal medicine services in the evolving coronavirus (COVID-19) pandemic • Using GDm-Health to monitor their blood glucose • Aged between 18 and 45 years • Willing and able to provide informed consent for participation in the study • Have and use a smartphone	• Multiple pregnancy • GDM not diagnosed by OGTT, HbA1C or fasting plasma glucose as defined by RCOG Guidance for maternal medicine services in the evolving coronavirus (COVID-19) pandemic • An absolute contra-indication to physical activity as per 2019 Canadian guidelines [25] e.g. preterm rupture of membranes, limited mobility, haemodynamically significant heart disease, restrictive lung disease • Unable to understand written or spoken English

*GDM* Gestational Diabetes Mellitus, *IADPSG* International Association of Diabetes and Pregnancy Study Groups, *RCOG*Royal College of Obstetrics and Gynaecology, *OGTT* Oral Glucose Tolerance Test

### Intervention

#### Visit 2: Motivational interview & smartphone app download

At visit 2, within 7 days of enrolment, participants received the + Stay-Active intervention. This involved attending a study visit conducted online (via the secure NHS online platform ‘Attend Anywhere’) or by telephone, depending on participant’s preference. During this visit, participants received a 20-min motivational interview with a trained research midwife and agreed on a set of weekly PA goals. Participants were also encouraged to download the ‘Stay-Active’ smartphone app and were shown the main features which include: recording their activities, reviewing their PA goals, and exploring the resource centre. Following the interview, participants completed the validated modified Oxford Maternity Diabetes Treatment Satisfaction Questionnaire (OMDTSQ) [26] (Supplement material 1) and were also asked to wear the accelerometer for a further week (total of 2 weeks) before returning it to the research team in the post.

All motivational interviews were audio recorded using a dictaphone (where participants consented to this). No patient identifiable data was recorded, the audio-file was labelled with a unique study specific number and transcriptions were de-identified. A randomly selected ten percent of motivational interview recordings were coded using the Motivational Interviewing Treatment Integrity Code (MITI 4.2.1) [27] by an experienced coder to assess the fidelity of the interview. MITI has two components: global summary scores (relational and technical dimensions) and behaviour counts. Global scores capture the coder’s overall impression of how well, or poorly, the interviewer performs in relation to the dimension being measured. Global scores are assigned to a five-point Likert scale with “1” being poor practice, “3” mixed practice, and “5” best practice. Behaviour counts are running tallies of the number of times a particular interview behaviour occurred and these are combined to give a further summary score. % Complex Reflection (%CR) is the percentage of total reflections which are judged complex (> 40% considered fair practice). A further summary of score for behaviour counts is the ratio of Reflections to Questions (R:Q): a 1:1 ratio is considered fair practice and 2:1 good practice.

Participants received a weekly telephone call from a member of the research team to review and adjust their activity goals. Participants were provided with individual motivational feedback messages from the research team at least weekly by text message via the Stay-Active app.

#### Follow-up assessment & completion of intervention

All participants were asked to attend a follow-up appointment at approximately 36 weeks’ gestation; during follow-up participants completed an online version of PPAQ [22], EVS [23] and OMDTSQ, and were provided with an accelerometer which they were asked to wear for 1 week before returning it to the research team by post. Participants were prompted to complete a feedback form on the intervention via the notifications on Stay-Active. The feedback form compromised of a 5- star rating system and free-text comment box to assess participants rating of goal setting, goal tracking, automated motivational messages and personalised messages about PA. A thematic analysis of the comments, and how these related back to behaviour change techniques and behaviour sources, was performed. Access to the Stay-Active app was terminated 1 week after the routine 36 weeks gestation follow-up appointment. Access was terminated as all data had been collected and support was no longer offered. The planned sample size was 60.

### Study outcomes

#### Primary outcomes

**Fig. 2:**
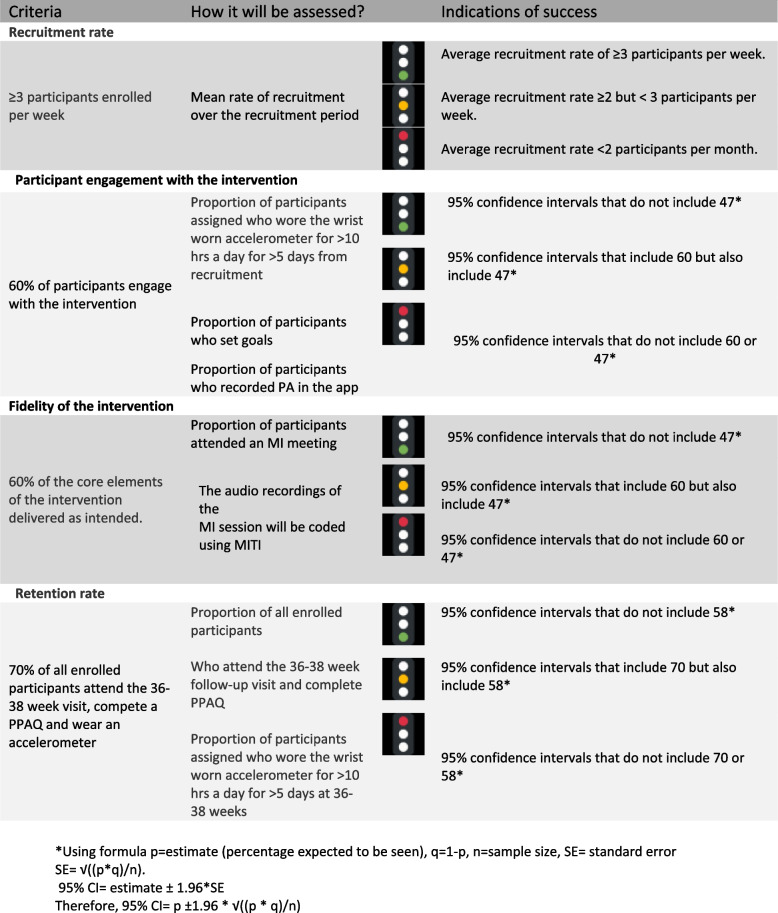
Primary outcome criteria

The primary outcomes were the feasibility and acceptability of the intervention to inform a decision on whether a subsequent randomised controlled trial is warranted. They were assessed against a set of predefined criteria (outlined in Fig. 2) related to (i) recruitment and (ii) retention rates, (iii) participant engagement with the intervention, (iv) acceptability and (iv) fidelity of the intervention. A traffic light system was used to determine the progression to a definitive trial. This system has been suggested to be preferable to the stop/go pass/fail approach [28]. The primary objectives with outcome measures, indications of success and timepoints are shown in Table 2 and Fig. 2.

**Table 2:** Objectives, outcome measures and timepoint of evaluation

**Objectives**	**Outcome measures**	**Timepoint(s) of evaluation of outcome measure**
**Primary objective**		
To evaluate how women with GDM interact, engage with and respond to *Stay-Active* + and to determine whether an RCT to assess the efficacy of this intervention is feasible	Recruitment rates • Percentage of eligible participants at the Gestational Diabetes Clinic, Women Centre, John Radcliffe Hospital • Percentage of women who fulfil the eligibility criteria and accept the invitation to participate	Recruitment & at end of study period
	Retention rate • Proportion of women that completed the study	At end of the study (36 weeks)
	Participant engagement with the intervention • Participant adherence rates to wrist worn accelerometer: ° Number of days worn over 7 days period, average daily wear, portion of wear; availability of data for PA outcome measures • Attendance rate at follow-up sessions • Completion rates of self-reported PA questionnaires • Proportion of participants who set goals on Stay-Active • Proportion of participants who recorded PA on Stay-Active	At visit 1& end of study period (36 weeks’ gestation)
	Acceptability: • Completion of the Oxford Maternity Diabetes Treatment Satisfaction Questionnaire (OMDTSQ) by participants	Visit 2 & end of study period (36 weeks gestation)
	Fidelity of the intervention • All Motivational Interviews will be audio recorded • 10% of motivational interviews will be coded using the Motivational Interviewing Treatment Integrity Code (MITI 4.2.1) to assess the fidelity of sessions	Visit 2 End of study period (36 weeks gestation)
**Secondary objectives**		
1. Assessment of PA	Attainment of information on physical activity time, type, intensity, and frequency assessed from baseline and subsequent visits i). Device specific (accelerometer) data: (Total PA average per measured day, moderate to vigorous PA and average Acceleration) ii). PPAQ – outcome: Energy expenditure iii). EVS – Weekly minutes of Moderate to Vigorous PA	At recruitment visit 2 and visit 3
2.Usage and Participant attitudes to + Stay-Active	i). Stay-Active Usage: • Average time spent on app per week • Average time per session • Frequency of app opened and duration per session • Number of participant logging activity per week ii). Participants attitudes to + Stay-Active (5 questions rating) on the usefulness of: Motivational interviewing, goal setting, tracking your goals via the app, automated motivational messages, personalised messages and an open comments section	From visit 2 to participant completion Visit 3: 36 weeks gestation
3. Assessment of blood glucose control & medication prescribed	i). Difference in glycaemic control measured as mean BG at recruitment and at 36–38 weeks (using BG taken in the week that the accelerometer is worn), adjusted for number and timing of measurements) ii). Participant’s prescribed medication (generic name and dose)	Recruitment & Visit 3 (36 weeks’ gestation)
4. Description of maternal and Neonatal outcomes	i). Maternal outcomes (weight gain, pharmacological medication (initiation, timing and doses in relation to meals and BG readings), hypertensive disorders of pregnancy (gestational hypertension and pre-eclampsia), gestation at delivery, mode of delivery) ii). Neonatal outcomes (birth weight, neonatal hypoglycaemia, neonatal hyperbilirubinaemia, admission to SCBU for > 24 h, shoulder dystocia)	Data gathered 6 weeks post delivery
5. Assessment of health costs	Number of additional visits, contacts made by research Midwife (both text message and telephone call) and time spent delivering intervention	Throughout study period
6 Determine any refinements required of the intervention	Review and analysis of the primary and secondary outcome data	Following data analysis

#### Secondary outcomes

Secondary outcomes include assessment of PA, usage, and participant attitudes to + Stay- Active; assessment of blood glucose measurements and control, description of maternal and neonatal outcomes, a description of additional health costs and any refinements required of the intervention (Table 2). Further details regarding the secondary outcome can be found in this study’s protocol publication [18].

### Statistics & analysis

The results consisted of descriptive statistics from assessments points. The statistics software packages Stata 14 (StataCorp, Texas, USA) and R (R Statistical Software (v4.1.2; R Core Team 2021) were used. Summary statistics were calculated for all measures. Continuous variables were reported as means, medians, standard deviations, percentiles (when appropriate), maximum and minimum values. Binary variables were reported as counts and percentages. The number of missing values were reported.

## Results

### Participants demographics

Sixty seven women enrolled in the study, with baseline demographics summarised in Table 3. The mean age of participants was 34 years and 52% were primiparous. Mean booking BMI was 30 kg/m^2^ and 65% of women classed themselves as of white ethnicity. Mean gestational age at recruitment was 27.5 weeks. Only one participant was taking pharmacological medication for GDM at recruitment.

**Table 3:** Demographics details of the participants

**Characteristic**	**N**	***mean (SD) or total (%)***
Maternal age (years)	67	33.6 (4.7)
Parity		
0	35	52.2%
1	17	25.4%
2	15	22.4%
*Total*	*67*	*100%*
BMI at booking (kg/m^2^)	66	30.0 (5.4)
First degree relative with diabetes		
No	32	47.8%
Yes	35	52.2%
*Total*	*67*	*100%*
Previous GDM^1^		
No	25	79.1%
Yes	7	21.9%
*Total*	*32*	*100%*
Previous baby weighing > 4.5kg^1^		
0	25	78.1%
1	6	18.8%
Unknown	1	3.1%
*Total*	*32*	*100%*
Previous Caesarean Section^1^		
No	22	68.8%
Yes	9	28.1%
Unknown	1	3.1%
*Total*	*32*	*100%*
Ethnic group		
White	46	68.7%
South Asian	8	11.9%
African/Caribbean	1	1.5%
East Asian	5	7.5%
Other	7	10.5%
*Total*	*67*	*100%*
OGTT (mmol/L)		
Fasting	67	5.01 (1.07)
2 h	67	8.21 (1.28)
Gestational age at recruitment (weeks)	67	27.5 (2.7)
Weight at recruitment (kg)	62	87.4 (15.7)
BP at recruitment (mmHg)		
SBP	67	110.2 (9.9)
DBP	67	64.8 (7.3)
No. of patients on pharmacological treatment (metformin ± insulin) at recruitment	1	1.5%

*BP* Blood pressure, *BMI* Body mass index*, GDM* Gestational Diabetes Mellitus*, OGTT* Oral Glucose Tolerance Test*, SBP* Systolic Blood Pressure*, DBP* Diasystolic Blood Pressure

^1^Multiparous women only

### Primary outcomes

#### Recruitment and retention rate

**Fig. 3:**
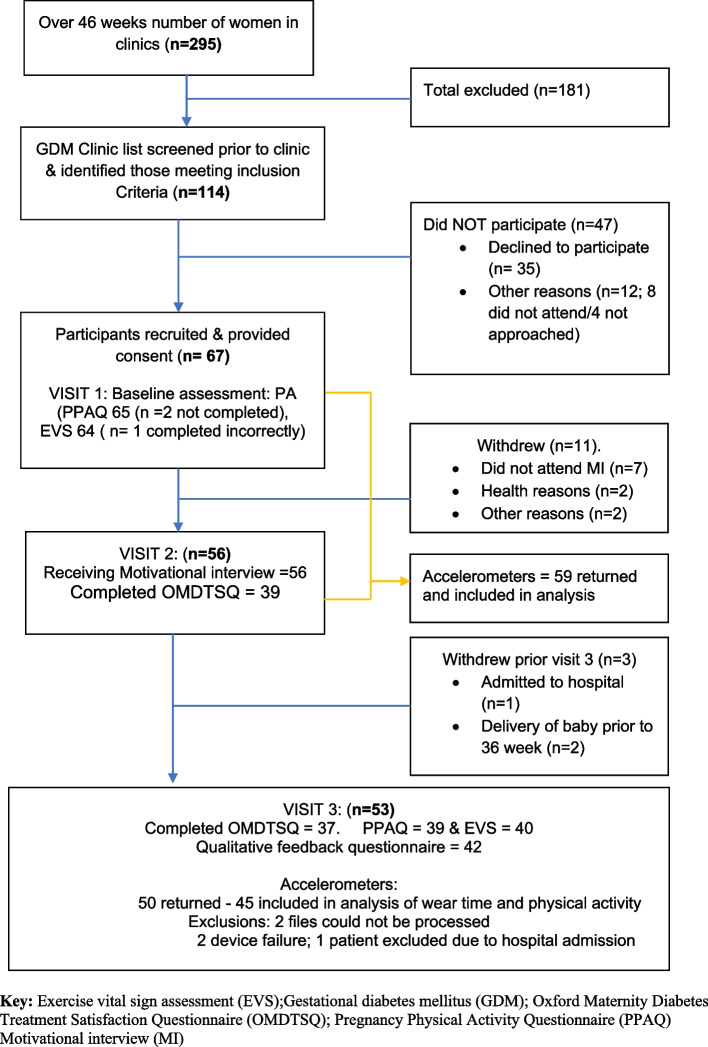
Flow chart of participants during the study

Over the 46-week recruitment period, 114 of 295 women met the inclusion criteria and 67 (58%) were enrolled in the study. Mean recruitment rate of 1.5 participants per clinic with 2.5 women per clinic meeting inclusion criteria (mean of 5.8 participant per month). Fifty-six (83%) received the intervention and 53 (79%) completed the study. Fourteen women (21%) who were enrolled in intervention did not complete the study. The majority of withdrawals (11 women) occurred prior to visit 2 with non-attendance to the online motivational interview being cited as the main reason. The three withdraws after visit 2 were related to health reasons (Fig. 3).

The results of the primary outcomes against the purposed traffic light system criteria are shown in Table 4. Recruitment rate only achieved a ‘red criteria’. Within the retention criteria, PPAQ completion and accelerometer wear achieved red and amber respectively. Once women had received the motivational interview; retention rate and engagement levels were high with 94% (53/56) completing the study and 98% (55/56) of participants set goals on Stay-Active In categories for participant engagement and fidelity of the intervention green criteria were achieved in all aspects and described in more detail below.

**Table 4:** Primary outcome variables with study findings and traffic light criteria

Variable	Assessment criteria with traffic light	Study findings:		Comment: (per protocol analysis was used for traffic light coding)
**Recruitment rate**				
Recruitment Rate (mean rate of recruitment)	Green: Average recruitment rate of ≥ 3 participants per week Yellow: Average recruitment rate ≥ 2 but < 3 participants per week Red: Average recruitment rate < 2 participants per month	Mean recruitment rate of 1.5 participants per clinic with 2.6 women per clinic meeting inclusion criteria		Red criteria: Limited by inclusion criteria and impact of COVID pandemic on the recruitment
**Participant engagement with the intervention**				
60% of participants engage with the intervention	Proportion of participants assigned who wore the wrist worn accelerometer for > 10 h a day for > 5 days from recruitment Proportion of participants who set goals Proportion of participants who recorded PA in the app	Per protocol	Intention to treat	Green Criteria All criteria achieved
		78% of participants (52/67) wore the device for more than 10 h on 5 or more days at baseline (95% CI 90, 66) 82% (55/67) of participants set goals on the Stay-Active. (95% CI 94, 70) 81% (54/67) submitted at least one PA record on the app (95% CI 93, 69	93% of participants (53/59) wearing the device for more than 10 h on 5 or more days at baseline 98%(55/56) of participants set goals on the Stay-Active 98% (54/55) submitted at least one PA record on the app	
**Fidelity of the intervention**				
60% of the core elements of the intervention delivered as intended	Proportion of participants attended an MI meeting The audio recordings of the MI session will be coded using MITI	83% (56/67) of participants received a motivational interview. All interviews were recorded. MITI 4.2 coding was performed for six motivational interviews chosen at random. (95% CI 95, 71)		Green Criteria All criteria achieved
**Retention rate**				
70% of all enrolled participants attend the 36-38 week visit, compete a PPAQ and wear an accelerometer	Proportion of all enrolled participants	53/67 (79%) women completed the intervention (95% CI 93, 71)		Green Criteria
	Assessment method:	Per Protocol	Intention to treat	
	Who attend the 36-38 week follow-up visit and complete PPAQ	39/67 (58%) completed the PPAQ (95% CI 69, 47)	39/53 (73%) completed the PPAQ	Red Criteria
	Proportion of participants assigned who wore the wrist worn accelerometer for > 10 h a day for > 5 days at 36–38 weeks	61% (41/67) wore the device for more than 10 h on 5 or more days at 36 weeks. (95% CI 72, 50)	85% (41/48) wearing the device for more than 10 h on 5 or more days at 36 weeks	Amber Criteria

*PPAQ* Pregnancy physical activity questionnaire (PPAQ), *PA* Physical activity, *COVID* Coronavirus (COVID-19)

#### Participant engagement with the intervention

Accelerometer data was available for analysis of accelerometer wear-time and PA levels for 59 participants invited to wear an accelerometer at baseline. Of these, 50 provided accelerometer data at 36 weeks, although 5 accelerometer files were not included in analyses of wear-time or physical activity levels. Two files could not be processed (likely device failure as data was not recorded). Three others were excluded: one was not worn at all during the measurement period, one returned implausibly high values for movement acceleration consistent with device malfunction, and one participant wore the device while an inpatient in hospital and thus it was considered that their data would not represent free-living compliance with measurement protocols or typical physical activity. Compliance to accelerometer measurement protocols was good with 78% of participants (52/67) wearing the device for more than 10 h on 5 or more days at baseline and 61% (41/67) at 36 weeks. Including only files available for analysis and adjusting for participants who had withdrawn, at 36 weeks, this rose to 91% (41/45). On average participants provided 6.1 (SD 2.0) valid days of accelerometer wear (> 10 h of wear) out of a possible 7 requested at baseline, and 6.0 (SD 2.1) at 36 weeks. Average daily accelerometer wear was 18.5 h per day (SD 7.4) at baseline and 17.2 h per day (SD 6.1) at 36 weeks (Tables 5 & 6).

**Table 5:** Physical activity data: self-reported physical activity results: exercise vital sign and pregnancy physical activity questionnaire

Exercise vital sign										
	mean	Median			Min		Max			SD
	Visit 1 (baseline) (64/67 completed)									
Minutes of moderate activity/ week	126	90			5		560			98.2
	VISIT 3 – 36 weeks (38/67 completed)									
Minutes of moderate/week	131	102.5			20		420			86.4
Pregnancy physical activity questionnaire (PPAQ)										
	mean	min	Percentiles					Max	SD	
			75th	50th		25th				
		BASELINE—65/67 questionnaires completed								
Total MET-hr/week	233.74	34.92	284.80	189.58		146.80		794	135.44	
Moderate activity MET-hr/week	78.09	0	79.46	41.25		18.21		540	96.16	
Vigorous MET-hr/Week	0.87	0	0.16	0.0		0.0		19.5	3.32	
		Visit 3 – 36 week (39/67 questionnaires completed)								
Total MET-hr/week	184.9	100	209.04	172.40		128.03		390	73.7	
Moderate activity MET-hr/week	46.2	1.67	49.86	27		20.50		207	49.7	
Vigorous MET-hr/Week	0.167	0	0	0		0		5.25	0.83

**Table 6:** Physical activity data:results of device specific (accelerometer) physical activity data

	Baseline (visit 1)	week 1(visit 2)	weeks 36 (visit 3)
Number of days worn over 7 day period for minimum 10 h	6.2 (1.8)	5.8 (1.9)	6.3 (2.3)
Proportion of days with 10 h wear (%)	88.3 (26.8)	84.0 (27.9)	91.0 (32. 1)
Number of days worn over 7 day period for minimum 16 h	5.4 (2.4)	4.8 (2.5)	5.1 (2.6)
Proportion of days with 16 h wear (%)	77.1 (34.4)	69.2 (3.5)	71.6 (3.7)
Number of days worn over 7 day period for minimum 24 h	4.1 (3.12)	3.6 (3.1)	3.4 (2.8)
Proportion of days with 24 h wear (%)	59.0 (44.0)	52.0 (44.0)	48.9 (3.9)
Average daily wear (hours per day)	18.45 (7.35)	17.59 (7.19)	16.65 (6.5)
Total PA minutes*	220.8 (80.8)	213.7 (83.7)	187.1 (64.2)
Moderate to Vigorous (MVPA) minutes*	50.2 (23.5)	46.2 (22.9)	42.1 (21.6)
Vigorous (VPA)*	3.3 (2.7)	3.13 (2.7)	2.9 (2.1)

^*^average per measured day on which an accelerometer was worn for > 10 h

(bracket number = Standard deviation)

There was high engagement with the intervention with 82% of participants (55/67) who engaged in the motivational interview (visit 2), setting goals on the Stay-Active app, and 98% (54/55) submitting at least one PA. Sustained engagement was evident with participants regularly accessing Stay-Active over multiple weeks (Table 7) and over 50% of participants continuing to access the app on a weekly basis until week 10. Completion rates of self-reported PA questionnaires were high; 97% of participants completed the PPAQ (65/67) and 95% the EVS (64/67) at visit 1. This was reduced at visit 3; with PPAQ rates at 58% (39/67) and EVS rates at 40/67 (59%), (Table 5). Accounting for withdrawn participants, this rose to 73% (39/53) and 75% (40/53) respectively.

**Table 7:** Weekly usage of stay-active app

**Week of study**	**Number of registered participants**	**Total number of app sessions**	**Mean number of app sessions per participant**	**Mean number of sessions accessing 'record my physical activity' per participant**	**Mean total time (seconds) spent on app per participant**	**Median duration (seconds) per app session (min, max)**	**Median duration (seconds) per session accessing 'record my physical activity' (min, max)**	**Proportion of participants accessing app**	**Proportion of participants accessing 'record my physical activity'**
1	55	493	9.0	4.2	341.7	23 (2, 487)	18 (2, 433)	98.2%	94.5%
2	55	312	5.7	2.7	139.1	18 (2, 276)	13 (2, 225)	90.9%	70.9%
3	55	347	6.3	2.3	154.1	16 (2, 398)	11 (2, 102)	92.7%	76.4%
4	55	281	5.1	2.0	100.9	15 (2, 135)	10 (2, 60)	83.6%	65.5%
5	55	264	4.8	2.2	107.3	15 (2, 163)	11 (2, 91)	80.0%	70.9%
6	55	250	4.5	2.0	103.1	15 (2, 260)	11 (2, 87)	80.0%	61.8%
7	54	244	4.5	1.4	87.6	13 (2, 181)	10 (2, 55)	70.4%	53.7%
8	50	190	3.8	1.5	73.2	14 (2, 172)	12 (2, 140)	74.0%	54.0%
9	49	152	3.1	1.0	67.6	15 (2, 151)	12 (3, 46)	61.2%	38.8%
10	44	135	3.1	1.2	53.6	13 (2, 125)	7 (3, 63)	56.8%	43.2%
11	42	102	2.4	0.8	47.7	12 (2, 144)	9 (3, 107)	52.4%	28.6%
12	35	68	1.9	0.7	37.8	13 (2, 80)	7 (4, 68)	45.7%	22.9%
13	22	22	1.0	0.2	17.6	12 (2, 89)	6 (5, 8)	36.4%	13.6%
14	17	18	1.1	0.2	12.9	12 (2, 33)	8 (6, 18)	29.4%	11.8%
15	8	7	0.9	0.4	12.3	14 (4, 26)	8 (8, 20)	37.5%	12.5%
16	2	3	1.5	1.0	17.5	13 (9, 13)	4 (4, 8)	50.0%	50.0%
17	1	6	6.0	5.0	154.0	13 (7, 73)	8 (8, 34)	100.0%	100.0%
18	1	2	2.0	2.0	36.0	17 (17, 19)	3 (3, 10)	100.0%	100.0%
19	1	2	2.0	2.0	26.0	12 (12, 14)	6 (6, 7)	100.0%	100.0%

#### Acceptability

The responses to the OMDTSQ indicated that women were strongly satisfied with their care throughout the study. Thirty-nine participants completed the questionnaire at visit 2 and 37 at visit 3. Supplement material 2 shows the satisfaction scores for each question demonstrating improvement in most domains particularly in PA specific questions at visit 3. Most participants favoured weekly feedback.

#### Fidelity of the motivational interviewing intervention

Fifty-six participants (83%) enrolled in the study received a motivational interview. All interviews were recorded. MITI 4.2 coding was performed for six motivational interviews chosen at random. Mean for relational global summary score was 3.25 (SD 0.27), technical global 3.17 (0.26), %CR 18% (14%), R:Q 0.66 (0.38). No interviews met any ‘good’ thresholds. ‘Fair’ thresholds were met in all six interviews for technical global summary score, three interviews for relational global summary score, and one interview for %CRand R:Q.

### Secondary outcomes

#### Physical activity assessment

Accelerometer defined PA levels across the study sample were very variable, and there was a trend for small reductions in activity between baseline and 36 weeks. On average total daily physical activity time (including light moderate and vigorous activity) reduced slightly between baseline (218.4 min per day [70.1]) and 36 weeks (195.8 min per day [64.2]). Daily moderate to vigorous activity also reduced between baseline (50.3 min per day [23.6])) and 36 weeks (43.9 min per day [22.1]) (Tables 5 & 6).

At baseline (visit 1); women reported a mean 78.09 ± 96.1 MET-hr/week moderate PA and vigorous PA of 0.87 ± 3.32 MET-hr/week (0.575 ± 2.49 MET-hr/week). For EVS; visit 1; the mean reported MVPA was 126 min per week (SD 98.2). At visit 3; PA levels were reduced mean 46.2 ± 49.7 MET-hr/week) and vigorous activity 0.1675 ± 0.837 MET-hr/week. However, EVS was higher than the mean reported MVPA at 131 (86.4) minutes per week.

#### Usage of stay-active

Fifty-five participants (82%) downloaded Stay-Active; it was used most frequently in the first six weeks of the intervention. For analysis, sessions with duration of less than two seconds were removed to reduce bias from accidental / compulsive opening of the app. In week 1; participants opened the application on average 9.0 times each, with a median duration per session of 23 (minimum 2, maximum 487) seconds. This was reduced by week 8, with 3.8 sessions per participant, and median duration of 14 (2, 172) seconds per session (Table 7). Participants logged a total of 699 physical activities (median 5 (1, 115) submissions per participant). In week 1, participants accessed the ‘record my physical activity’ section of the app on average 4.2 times each (median duration 18 (2, 433) seconds per session), this reduced to 1.5 times in week 8 (median duration 12 (2, 140) seconds). Thirty participants accessed the resource centre at least once spending an average time of 21.5 s per session.

Forty-three participants completed feedback on +Stay-Active. On a five-star rating scale (0 worst, 5 best), the percentage of participants rating the motivational interview as four or five stars was 95.3%, goal setting 97.7%, goal tracking 88.4%, automated motivational messages 76.7% and personalised messages about physical activity 93%.

The most common feedback from the free-text comments was that the app was easy to use. Suggestions on how to improve the functionality of the app were also frequent and centred around being able to record physical activity in more detail and review previous activity. Free-text comments (Table 8) demonstrated the effect of the app on a variety of behaviour change techniques and behaviour sources including psychological capability, reflective and automatic motivation [16].

**Table 8:** Overview of thematic analysis of free-text feedback

**Example comment**	**Theme of comment / BCT**	**Behaviour source targeted**
*“Good to see progress in the app with regards to goals achieved”* *“The app is good to track what I'm doing”* *“I would like to look back on recorded activity (what I did when”)*	Set, monitor, and review physical activity goals Goal setting [1.1] Review behaviour goals [1.5] Self-monitoring of behaviour [2.3] Prompts and cues [7.1]	Psychological capacity Reflective motivation
*“I have found the discussions and app both increasing my motivation”* *“I found the app useful, easy to use and motivating.”*	Increase in motivation	Reflective motivation
*“The messages and the notifications are a real 'keep going, you got this' message”* *“I find the messages and the notifications very motivating and offers support to continue going”* *“As I reached 36 weeks I found the automated messages less motivational as I was finding it more difficult to carry out physical activity”* *“Personally feel like the motivation isn’t needed as those messages don’t have any impact for me”*	Mixed views on messages and notifications Feedback on behaviour [2.2] Prompts and cues [7.1]	Psychological capacity Reflective motivation Automatic motivation

*BCT* Behaviour change technique

[bracketed numbers] refers to the Behaviour Change Technique Taxonomy v1

#### Assessment of blood glucose control

Over the period of enrolment, mean blood glucose fell. In the first week after recruitment (at a mean gestation of 28 weeks) the mean blood glucose was 6.3 mmol/l which reduced to 6.1 mmol/l for the week after the Motivational interview intervention (at a mean gestation of 29 weeks) and reduced further to 5.8 mmol/l at a mean gestation of 36 weeks. This represents a change between these time points of: -0.16 (mean gestational week 28 and 29), -0.54 (mean gestational week 28 and 36) and -0.30 (mean gestational week 29 and 36). This was accounted for by a fall in both the fasting and postprandial blood glucose values (Supplement material 3).

#### Description of maternal and neonatal outcomes

Outcome data on 59 mother-baby pairs was available. Mean gestational age at birth was 39.2 weeks. 14% of women had a planned caesarean section (CS), 39% of women had an unassisted vaginal birth, 32% had an emergency CS and 15% of women had an assisted vaginal birth. Thirty nine percent of women had post-partum bleeding of more than 500mls, one woman had major perineal trauma, nine women had a hypertensive disorder of pregnancy, and no women required admission to the intensive care unit. There was a mean on 0.82 kg maternal weight gain between recruitment and last recorded weight before birth representing a mean of 0.06 kg weight gain per week.

The mean birth weight was 3401 g, with eight babies having a birth weight above 90th centile. 58% were female. No shoulder dystocia or neonatal hypoglycaemia requiring treatment was reported. Three babies had hyperbilirubinaemia and one had birth trauma. Four babies required admission to the neonatal intensive care for a mean duration of 1.6 days. These adverse outcomes were assessed and found to be not related to the intervention (Supplement material 4).

#### Assessment of participants contacts

A total of 367 follow-up phone calls were made to participants during the study. Seventy percentage (259) were answered by participants. A total of 959 motivational SMS messages were sent from Stay-Active. An additional 75 messages were sent for the initial setup credentials and forgot password requests.

## Discussion

This study is the first to explore the feasibility and acceptability of this combined intervention aimed at maintaining PA levels in women with GDM. All indicators of success were achieved within the categories for participant engagement and fidelity of the intervention, nevertheless not all were fulfilled within recruitment and retention rates. The recruitment rate was lower than expected and the mean number of participants meeting the eligibility criteria was only 2.5 participants/week; the most likely explanation is the reduction in face-to-face consultations during the COVID pandemic. An assessment of future clinical activity and the proportion of women meeting the eligibility criteria would be prudent. Once participants received the motivational interview; they appeared to remain actively engaged in the study but future considerations will be given to maximising participants attendance at this visit.

This study adds to the literature regarding the development of a complex PA intervention to aid the wider management of GDM. Management involves counselling, dietary modification, PA, glucose monitoring, and supplemental pharmacological therapies. The implementation of individual management elements vary. GDM specific smartphone apps can provide an opportunity to improve management. A systematic review of the effectiveness of mobile health applications for GDM included five RCTs and found improved trends in glycemic control, pregnancy and birth related outcomes [29]. The Apps support women with automatic transfer of blood glucose values from a glucometer to their smartphone and onwards to the supporting healthcare team, and some provided varying tailored lifestyle information on diet, PA, breastfeeding and GDM [30, 31]. Similar to + Stay-Active feedback, studies have described that these smartphone apps are appealing to women with overwhelmingly positive feedback [32]. Whilst positive results have been reported in improved compliance of blood glucose monitoring [33, 34], significantly lower blood glucose measurement and lower rate of insulin needed [34]; smartphone-based apps alone have not been clearly shown to improve pregnancy outcomes [35]. Immanuel and Simmons highlight that many studies [15, 31, 34] have been underpowered to detect improvement in pregnancy outcomes [35]. Furthermore, the specific content, measurement or analysis of any PA interventions were limited or not reported [15, 30, 31]. Our work provides a step forward in delivering, measuring, and analysing a specific PA intervention for this population.

Adherence to accelerometer measurement protocols were excellent, with moderate levels of completion rates of self-reported PA assessments and satisfaction questionnaires. This may reflect burden of the high number of questionnaires participants were expected to complete. This could be refined and re-enforces the capability to capture this data in our population.

The evidence supporting the benefits of PA among women with GDM is mounting. A further metanalysis published in 2022; concluded exercise intervention can improve the blood glucose parameters and can also reduce adverse pregnancy outcomes, such as premature birth and macrosomia [36]. This supports separate analyses that found requirements of insulin therapy, dosage and latency to administration were improved in the exercise intervention groups [8, 37]. However, most exercise interventions are supervised and well resourced; potentially being difficult to translate into the healthcare setting. Integration of health coaching and evidence based behavioural strategies (goal setting, monitor and feedback) may provide the most appropriate tools for translation of this evidence into clinical practice [38]. Multicomponent PA interventions appear to be more effective than standalone interventions [39, 40]. In our study, Women responded positively to the combination of motivational interviewing and support through Stay-Active. Re-enforcing this, is promising results from a randomised trial, that used a similar approach to + Stay-Active, found the combination of a mobile phone app and brief counselling increased objectively measured PA over 3 months in physically inactive non-pregnant women [41]. This combined approach has successfully been used to enhance the daily level of PA among older adults [42]. Within pregnant women,,motivational interviewing was found to improve adherence to healthy eating in addition to routine care in women with type 2 diabetes [43] and in a recent prospective RCT involving online health-coaching led to women increasing or at least maintaining their level of PA during the course of their pregnancy [44]. Furthermore, Smartphone apps have been found to be effective for increasing objectively measured PA in pregnant women [45].

The timing of our intervention was essential, building on a potential ‘teachable moment’ [46] following a diagnosis of GDM where there is opportunity for women to re-focus on PA with the health of the baby and glycaemic control being strong motivators. Potentially, optimising the effect of motivational interview.

Sustained engagement was evident with participants regularly accessing the Stay-Active app and logging activity for multiple weeks. The gradual reduction in the number of sessions and time spent on the app may represent increased familiarity of participants with the app and the effect of behaviour change, or disengagement. Evidence of sustained engagement is important, and not always evident. For example, in a large RCTs (*n* = 170 in each arm) to evaluate the effects of a smartphone app–based lifestyle coaching program ((Habit-GDM) a program comprised 12 interactive lessons); only 49.4% of the intervention women accessed the educational lessons [30]. In another multicentre nested randomised trial involved 162 pregnant; whereby 77 women (77/162) in addition to lifestyle advice were provided with access to a smartphone application designed to encourage women to set dietary and PA goals and monitor their progress only 24 women (31.2%) reported using the smartphone application [47]. Motivational interviewing together with regular follow up and individualised reminder messages, helped maintain engagement over the study period. We feel there is the unique opportunity for clinicians to play a key role by interacting and supporting the service user via Stay-Active.

With the increasing number of women with GDM and greater pressures on health care providers to streamline services; digital technologies are expected to provide remote support at scale. Nevertheless, during our study support was considerable with motivational inteviews, regular telephone follow ups and over 900 text messages sent; the effectiveness on clinical outcomes will need to be balanced with intervention and implementation costs. More robust resource utilization and cost-effective analysis within GDM App studies is required [32] and needs to be consider in future work.

The study demonstrated moderate acceptability for the fidelity of motivational interviewing. with the complex reflections and ratio of reflection to questions were generally below ‘fair’ proficiency. This highlights motivational interviewing is a challenging skill. Multifaceted training, practice and mentoring would be required to meet the accepted proficiency thresholds in the future.

The participant characteristics were typical of this single centre and with only one participant on pharmacological medication for GDM at recruitment. Glycaemia control improved over the study period as one would expect as all women received active clinical management of their GDM. It is encouraging to see this improvement and we can conclude that the addition potential burden of this intervention did not adversely affect glycaemic control. Maternal and neonatal outcomes were also broadly as expected. Weekly weight gain was only 0.06 kg during the study period, 48% of women were on pharmacological treatment at birth which compares with historical cohorts in the same centre and suggests the intervention was not associated with a reduction in the need for pharmacological medication**.**

Both the PPAQ and objective accelerometer demonstrated a reduction of MVPA by visit 3; however, this is expected with activity levels typically declining during pregnancy [48]. Due to the lack of a control group, drawing conclusions regarding the impact of this intervention on the rate of decline in PA level is not possible. Additionally, there is no normative data for PPAQ within UK populations, and due to variations in methodology and study population, it is difficult to compare activity levels as measured by PPAQ between studies [49]. Within the PPAQ data set in particular, there were a small minority of outlying values with very high PA levels reported. Despite their practicality, it is an established limitation of PA self-reported data that they are subject to significant error and bias. Recalling and reporting PA is challenging, often leading to participants over or under-reporting PA. The resultant misclassification can impact the ability to detect associations or intervention-related behaviour change. With the higher levels of adherence to accelerometer measurement protocols and lower completion rates of the self-reported questionnaires; our further work would focus on using this objective measure of PA.

We believe that a larger multi-centre randomised controlled trial to investigate the effectiveness of this intervention is now warranted. Prior to this, further training is required to ensure motivation interviewing meets the accepted proficiency thresholds. Inclusion criteria should be reviewed to optimise participant recruitment and clinic activity assessed. This study demonstrated this combined behavioural change driven approach maintained high levels of engagement. There is already a commercially available CE-marked smartphone glucose management application GDm-Health [15] embedded within the clinical pathway for women with GDM at the study site, which has previously shown high levels of patient engagement, compliance and usage [26]. Given that + Stay-Active was found to be feasible and acceptable, an additional functionality to apps such as GDm-Health could be considered, improving usability and accessibility allowing users to observe the direct impact of PA of their blood glucose control.

Further work to assess whether this intervention model could be transferred to other populations of pregnant women or non-pregnant patients with comorbidities to evaulated PA and clinical outcomes, is required.

### Strengths and limitations of this study

This study used several outcomes to provide evidence on the feasibility and acceptability of this complex intervention. However, the study design and size was not powered to determine intervention efficacy or clinical effectiveness. It was within a single centre, non-randomised and lacked a control group. The study partially recruited during the COVID-19 pandemic, meaning that interviews were remote and opportunities for exercise outside the home may have been limited for some women. Therefore, conclusions cannot be drawn regarding effectiveness of the intervention. Participation was not mandatory, which may have resulted in a selection bias towards those who have a tendency/preference to undertake higher levels of PA.

## Conclusions

The delivery of this combined intervention designed to support PA in pregnant women was feasible and well accepted. Recruitment rate was lower than expected and affected by the COVID pandemic. Retention rates were satisfactory and there was a high level of participant compliance with PA measurements and engagement throughout the study. A future RCT to explore the efficacy of this intervention to increase PA and evaluate the effect on clinical outcomes would be feasible.

### Supplementary Information

**Supplementary Material 1.**

**Supplementary Material 2.**

**Supplementary Material 3.**

**Supplementary Material 4.**

## Data Availability

The datasets generated and analysed during the current study are not publicly available due arrangements specified in ethics approval, but anonymised data is available from the corresponding author on reasonable request.

## Abbreviations

App: Application
BCTs: Behaviour Change Techniques
BCW: Behaviour Change Wheel
EVS: Exercise vital sign assessment
GDM: Gestational diabetes mellitus
MVPA: Moderate to vigorous physical activity
NICE: National Institute for Health and Care Excellence
OMDTSQ: Oxford Maternity Diabetes Treatment Satisfaction Questionnaire
%CR: Percentage Complex Reflections
PA: Physical activity
PPAQ: Pregnancy Physical Activity Questionnaire
RCT: Randomised control trial
R:Q: Reflection to Question ratio
SD: Standard deviation
