# Supplementary material for: A feasibility study using motivational interviewing and a smartphone application to promote physical activity (+Stay-Active) for women with gestational diabetes
Source: BMC Pregnancy Childbirth. 2024 May 14;24:360. doi: 10.1186/s12884-024-06508-w (PMC11094872; doi:10.1186/s12884-024-06508-w)
Supplement: Supplementary file 1 — Supplementary Material 1. [file 12884_2024_6508_MOESM1_ESM.pdf]

Oxford Maternity Diabetes Treatment Satisfaction Questionnaire (GDM Health & Stay active)

Please indicate your personal agreement with each of the following statements

* Required

1. Please enter your study participant number *

2. Visit 1 or 2 *

   ◯ Visit 1(initial consultation)

   ◯ Visit 2 (approx 36 weeks)

3. I find the equipment I use to check my blood sugars is convenient *

   ◯ Strongly agree

   ◯ Agree

   ◯ Neutral

   ◯ Disagree

   ◯ Strongly disagree

   ◯ N/a

4. I feel the equipment I use to check my blood sugars is reliable *

- ◯ Strongly Agree
- ◯ Agree
- ◯ Neutral
- ◯ Disagree
- ◯ Strongly disagree
- ◯ N/A

5. My blood sugar monitoring fits in with my lifestyle *

- ◯ Strongly Agree
- ◯ Agree
- ◯ Neutral
- ◯ Disagree
- ◯ Strongly disagree
- ◯ N/A

6. The feedback I receive about my blood sugar level is useful *

- ◯ Strongly Agree
- ◯ Agree
- ◯ Neutral
- ◯ Disagree
- ◯ Strongly disagree
- ◯ N/a

7. I feel the system I use to calculate carbohydrate is convenient *

- ◯ Strongly agree
- ◯ Agree
- ◯ Neutral
- ◯ Disagree
- ◯ Strongly disagree
- ◯ N/A

8. I feel the system I use to calculate carbohydrate is reliable *

- ◯ Strongly agree
- ◯ Agree
- ◯ Neutral
- ◯ Disagree
- ◯ Strongly disagree
- ◯ N/a

9. I feel the feedback I receive about my carbohydrate intake is useful *

- ◯ Strongly agree
- ◯ Agree
- ◯ Neutral
- ◯ Disagree
- ◯ Strongly disagree
- ◯ N/A

10. I feel the system I use to record my weight is convenient *

- Strongly agree
- Agree
- Neutral
- Disagree
- Strongly disagree
- N/A

11. I feel the system I use to record my weight is useful *

- Strongly agree
- Agree
- Neutral
- Disagree
- Strongly disagree
- N/A

12. I feel the system I use to measure my physical activity/exercise level is convenient *

- Strongly agree
- Agree
- Neutral
- Disagree
- Strongly disagree
- N/A

13. I feel the feedback I receive about my physical activity/exercise levels is useful *

- ◯ Strongly agree
- ◯ Agree
- ◯ Neutral
- ◯ Disagree
- ◯ Strongly disagree
- ◯ N/A

14. How often would you have liked feedback? *

- ◯ Daily
- ◯ Every 2-3 days
- ◯ Every 4-5 days
- ◯ Weekly
- ◯ Only when necessary
- ◯ N/A

15. Is there a particular area where you would have liked more feedback? *

- ◯ Blood glucose
- ◯ Carbohydrate intake
- ◯ Physical Activity/Exercise
- ◯ Weight gain
- ◯ None

16. Please use box below for any further comments: Particular regarding the Stay-Active App (ease of use & recommendation to the others)

This content is neither created nor endorsed by Microsoft. The data you submit will be sent to the form owner.

Microsoft Forms
