# Supplementary material for: A feasibility study using motivational interviewing and a smartphone application to promote physical activity (+Stay-Active) for women with gestational diabetes
Source: BMC Pregnancy Childbirth. 2024 May 14;24:360. doi: 10.1186/s12884-024-06508-w (PMC11094872; doi:10.1186/s12884-024-06508-w)
Supplement: Supplementary file 2 — Supplementary Material 2. [file 12884_2024_6508_MOESM2_ESM.docx]

Supplement 2: Oxford Maternity Diabetes Treatment Satisfaction Questionnaire results (Visit 2&3)

| Visit 2 (39 responders) | N/A | Strongly Disagree | Disagree | Neutral | Agree | Strongly agree | Satisfaction score |
| --- | --- | --- | --- | --- | --- | --- | --- |
|  |  | (-2) | (-1) | 0 | (+1) | (+2) |  |
| I find the equipment I use to check my blood sugars is convenient | 0 | 0 | 0 | 2 | 22 | 15 | 52 |
| I feel the equipment I use to check my blood sugars is reliable | 0 | 0 | 0 | 2 | 25 | 12 | 49 |
|  |  |  |  |  |  |  |  |
| My blood sugar monitoring fits in with my lifestyle | 0 | 0 | 4 | 11 | 19 | 5 | 25 |
| The feedback I receive about my blood sugar level is useful | 0 | 0 | 2 | 4 | 22 | 11 | 42 |
|  |  |  |  |  |  |  |  |
| I feel the system I use to calculate carbohydrate is convenient | 23 | 0 | 1 | 3 | 9 | 3 | 14 |
| I feel the system I use to calculate carbohydrate is reliable | 24 | 0 | 1 | 3 | 8 | 3 | 13 |
| I feel the feedback I receive about my carbohydrate intake is useful | 21 | 0 | 5 | 5 | 6 | 2 | 5 |
|  |  |  |  |  |  |  |  |
| I feel the system I use to record my weight is convenient | 27 | 0 | 1 | 2 | 7 | 2 | 10 |
| I feel the system I use to record my weight is useful | 28 | 0 | 1 | 2 | 6 | 2 | 9 |
|  |  |  |  |  |  |  |  |
| I feel the system I use to measure my physical activity/exercise level is convenient | 4 | 0 | 1 | 3 | 24 | 7 | 37 |
| I feel the feedback I receive about my physical activity/exercise levels is useful | 4 | 0 | 1 | 3 | 21 | 10 | 40 |
|  |  |  |  |  |  |  |  |
|  |  | Daily | Every 2-3 days | Every 4-5 days | Weekly | Only when necessary |  |
| How often would you have liked feedback? |  | 0 | 5 | 3 | 21 | 8 |  |
|  |  | Blood glucose | Carbohydrate intake | Physical Activity/Exercise | Weight gain | None |  |
| Is there a particular area where you would have liked more feedback? |  | 5 | 13 | 2 | 3 | 16 |  |

| Visit 3 (37 responders) | N/A | Strongly Disagree | Disagree | Neutral | Agree | Strongly agree | Satisfaction score | Difference from visit 1 |
| --- | --- | --- | --- | --- | --- | --- | --- | --- |
|  |  | (-2) | (-1) | 0 | (+1) | (+2) |  |  |
| I find the equipment I use to check my blood sugars is convenient | 0 | 0 | 1 | 3 | 14 | 19 | 51 | -1 |
| I feel the equipment I use to check my blood sugars is reliable | 0 | 0 | 3 | 3 | 15 | 16 | 44 | -5 |
|  |  |  |  |  |  |  |  |  |
| My blood sugar monitoring fits in with my lifestyle | 0 | 1 | 3 | 6 | 18 | 9 | 31 | 6 |
| The feedback I receive about my blood sugar level is useful | 0 | 0 | 1 | 3 | 19 | 14 | 46 | 4 |
|  |  |  |  |  |  |  |  |  |
| I feel the system I use to calculate carbohydrate is convenient | 17 | 0 | 1 | 8 | 7 | 4 | 14 | 0 |
| I feel the system I use to calculate carbohydrate is reliable | 17 | 0 | 0 | 11 | 5 | 4 | 13 | 0 |
| I feel the feedback I receive about my carbohydrate intake is useful | 21 | 0 | 0 | 8 | 4 | 4 | 12 | 7 |
|  |  |  |  |  |  |  |  |  |
| I feel the system I use to record my weight is convenient | 21 | 0 | 0 | 5 | 6 | 5 | 16 | 6 |
| I feel the system I use to record my weight is useful | 21 | 0 | 0 | 5 | 6 | 5 | 16 | 7 |
|  |  |  |  |  |  |  |  |  |
| I feel the system I use to measure my physical activity/exercise level is convenient | 0 | 0 | 0 | 6 | 23 | 8 | 39 | 2 |
| I feel the feedback I receive about my physical activity/exercise levels is useful | 0 | 0 | 0 | 5 | 22 | 10 | 42 | 2 |
|  |  |  |  |  |  |  |  |  |
|  |  | Daily | Every 2-3 days | Every 4-5 days | Weekly | Only when necessary |  |  |
| How often would you have liked feedback? |  | 3 | 4 | 3 | 20 | 7 |  |  |
|  |  | Blood glucose | Carbohydrate intake | Physical Activity/Exercise | Weight gain | None |  |  |
| Is there a particular area where you would have liked more feedback? |  | 4 | 8 | 2 | 3 | 20 |  |  |
