# Supplementary material for: A feasibility study using motivational interviewing and a smartphone application to promote physical activity (+Stay-Active) for women with gestational diabetes
Source: BMC Pregnancy Childbirth. 2024 May 14;24:360. doi: 10.1186/s12884-024-06508-w (PMC11094872; doi:10.1186/s12884-024-06508-w)
Supplement: Supplementary file 3 — Supplementary Material 3. [file 12884_2024_6508_MOESM3_ESM.docx]

*Supplement 3. Secondary Outcomes: Mean Blood Glucose excluding participants who withdrew*

|  | **1st week after recruitment (pre-MI)** | **2^nd^ week after recruitment (post MI)** | **T3** |
| --- | --- | --- | --- |
| **Gestation**  **n**  **Mean (SD)** | 57  27.6 (2.8) | 57  28.6 (2.8) | 54  36.0 (0.6) |
| **Blood glucose**  **Fasting**  **n**  **Mean (SD)**  **Postprandial**  **n**  **Mean (SD)**  **Total**  **n**  **Mean (SD)** | 57  5.1 (0.6)  57  6.7 (1.0)  57  6.3 (0.8) | 53  5.1 (0.5)  54  6.5 (0.7)  54  6.1 (0.6) | 54  4.7 (0.3)  54  6.3 (0.6)  55  5.8 (0.5) |
|  | **W2 vs. W1** | **T3 vs. W1** | **T3 vs. W2** |
| **Change in fasting blood glucose**  **n**  **Mean (95% CI) ^1^**  **Change in postprandial blood glucose**  **n**  **Mean (95% CI)**  **Change in total blood glucose**  **n**  **Mean (95% CI)** | 53  -0.05 (-0.16, 0.05)  54  -0.11 (-0.34, 0.12)  54  -0.16 (-0.34, 0.02) | 53  -0.38 (-0.52, -0.23)  53  -0.45 (-0.71, 0.20)  54  -0.54 (-0.76, -0.33) | 50  -0.31 (-0.44, -0.19)  51  -0.27 (-0.48, -0.06)  51  -0.30 (-0.47, -0.12) |

^1^ change in blood glucose (95% CI) accounting for number of patients and number of observations at each time point

MI – motivational interviewing CI – confidence interval SD – Standard deviation

W1 - week 1; W2- week 2; T3 – final visit (visit 3)
