# Supplementary material for: A feasibility study using motivational interviewing and a smartphone application to promote physical activity (+Stay-Active) for women with gestational diabetes
Source: BMC Pregnancy Childbirth. 2024 May 14;24:360. doi: 10.1186/s12884-024-06508-w (PMC11094872; doi:10.1186/s12884-024-06508-w)
Supplement: Supplementary file 4 — Supplementary Material 4. [file 12884_2024_6508_MOESM4_ESM.docx]

*Supplement 4. Maternal and Neonatal clinical outcomes excluding participants who withdrew*

| **Outcome** | **N** | **n (%) or mean (SD)** |
| --- | --- | --- |
| Maternal PET present  No  Yes  *Total* | 56  3  *59* | 94.9%  5.1%  *100%* |
| Gestational age at delivery in weeks | 59 | 39.2 (1.2) |
| Mode of delivery  SVD  Vacuum  Forceps  Planned C/S  Emergency C/S  *Total* | 23  1  8  8  19  *59* | 39.0%  1.7%  13.6%  13.6%  32.2%  *100%* |
| Major perineal trauma  No  Yes  *Total* | 58  1  *59* | 98.3%  1.7%  *100%* |
| PPH>500ml  No  Yes  *Total* | 36  23  *59* | 61.0%  39.0%  *100%* |
| Other maternal delivery conditions  No  Yes – Unknown  OC ferinject for low Hb  Blood transfusion  Low Hb  Mild ITP  Sent home with catherter & hypertens  Sepsis  2.2L blood loss, return to theatre  GBS pos  MOH  MOH, BT, blood patch  Obstetric cholestasis  Vaginal pack  *Total* | 45  1  1  1  1  1  1  3  0  0  1  1  1  1  *59* | 76.3%  1.7%  1.7%  1.7%  1.7%  1.7%  1.7%  5.1%  0%  0%  1.7%  1.7%  1.7%  1.7%  *100%* |
| Last recorded weight (kg) | 51 | 84.7 (14.2) |
| Gestation at last recorded weight in weeks | 51 | 33.9 (6.4) |
| Total weight gain between recruitment and last recorded weight (kg) | 47 | 0.82 (4.08) |
| Weight gain per week between recruitment and last recorded weight (kg/week) | 47 | 0.06 (0.32) |
| Maternal pregnancy-induced hypertension or preeclampsia  No  Yes  *Total* | 50  9  *59* | 84.8%  15.2%  *100%* |
| Admission to higher level of care for mother (nights) | 66 | 0 (0) |
| Hypoglycaemic medication at delivery (excluding participants who withdrew)  No  Insulin only  Metformin only  Insulin and Metformin  *Total* | 29  2  17  8  *56* | 51.8%  3.6%  30.4%  14.3%  *100%* |
| Delivery less than 37 weeks  ≥ 37 weeks  < 37 weeks  *Total* | 57  2  *59* | 96.6%  3.4%  *100%* |
| Birth weight in grams (sd) | 58 | 3400.9 (503.3) |
| Birth weight centile (median, IQR) | 58 | 3485 (q25 3065, q75 3750) |
| Birth weight centile > 90 | 8 | 3985 (p90) |
| Sex  Male  Female  Unknown  *Total* | 24  34  1  *59* | 40.7%  57.6%  1.7%  *100%* |
| Shoulder dystocia  No  Yes  Unknown  *Total* | 58  0  1  *59* | 98.3%  0%  1.7%  *100%* |
| Birth trauma  No  Yes  Unknown  *Total* | 57  1  1  *59* | 96.6%  1.7%  1.7%  *100%* |
| APGAR Score  7  8  9  10  Unknown  *Total* | 1  0  5  52  1  *59* | 1.7%  0%  8.5%  88.1%  1.7%  *100%* |
| Neonatal hypoglycaemia (requiring IV glucose)  0  10  Unknown  *Total* | 58  0  1  *59* | 98.3%  0%  1.7%  *100%* |
| Neonatal hyperbilirubinaemia  No  Yes  Unknown  *Total* | 55  3  1  *59* | 93.2%  5.1%  1.7%  *100%* |
| Other neonatal conditions  None  IFT for oral assessment  PROM  PROM, PREM  SGA  Significant MEC  Transposition of the great arteries  Grunting  Low sats  Sepsis  Sepsis IV Abx  *Total* | 49  1  0  1  1  2  1  1  1  1  1  *67* | 83.1%  1.7%  0%  1.7%  1.7%  3.4%  1.7%  1.7%  1.7%  1.7%  1.7%  *100%* |
| Admission to higher level of care for baby | 4 | 6.0% |
| Duration of neonatal stay | 58 | 1.6 (1.1) |
| Admission to SCBU  No  Yes  Unknown  *Total* | 54  4  1  *59* | 91.5%  6.8%  1.7%  *100%* |
| SCBU night stayed  0  1  2  Unknown  *Total* | 54  0  1  4  *59* | 91.5%  0%  1.7%  6.8%  *100%* |

Key:

PET -Pre-eclampsia; SVD – Spontaneous vaginal delivery. C/S – Caesarean section

PPH - Postpartum haemorrhage; Hb- haemoglobin; ITP- Immune thrombocytopenic purpura; GBS -Group B Streptococcus; MOH - Massive Obstetric Haemorrage; PROM- Premature rupture of membranes; MEC - meconium aspiration syndrome; SGA- Small for gestational age (SGA) Sats- Oxygen saturations; SCBU - Special care baby unit IV- intravenous; IFT- infant feeding team.
